# Carbon pricing, co-pollutants, and climate policy: Evidence from California

**DOI:** 10.1371/journal.pmed.1002610

**Published:** 2018-07-17

**Authors:** James K. Boyce, Michael Ash

**Affiliations:** 1 Department of Economics and Political Economy Research Institute, University of Massachusetts Amherst, Amherst, Massachusetts, United States of America; 2 Department of Economics and School of Public Policy, University of Massachusetts Amherst, Amherst, Massachusetts, United States of America

## Abstract

In a Perspective, James Boyce and Michael Ash discuss Lara Cushing and colleagues' research study on the implications of California's policy on carbon trading.

Carbon pricing policies, a critical instrument to manage the costs of greenhouse gas (GHG) pollution, now cover roughly 20% of the world's carbon emissions from fossil fuel combustion [1]. By increasing the price of fossil fuels via a carbon tax or a cap-and-permit system, these policies reduce their use and provide incentives for investments in alternative energy sources and energy efficiency.

Carbon pricing can also yield public health co-benefits by reducing emissions of hazardous air co-pollutants along with carbon dioxide and can, in principle, improve environmental equity by reducing disproportionate pollution burdens in socioeconomically disadvantaged communities. In this issue of *PLOS Medicine*, Rachel Morello-Frosch and colleagues report the first evidence-based assessment of air pollution equity outcomes of California’s carbon pricing policy implemented in 2013. They found that, over the 2011–2015 study period, emissions from industrial facilities of GHGs and co-pollutants—particulate matter (PM_2.5_), sulfur oxides, nitrogen oxides, volatile organic compounds (VOCs), and air toxics (roughly 600 chemicals subject to Toxics Release Inventory reporting)—were almost as likely to increase as to decrease and that neighborhoods with emission increases tended to have higher percentages of racial and ethnic minority, poor, less educated, and linguistically isolated residents [2]. These findings suggest that climate policy can best fulfill its potential if designed explicitly to achieve widely shared health co-benefits and improved environmental equity.

## Co-pollutants and climate policy

The World Health Organization estimated the burden of disease attributable to ambient air pollution at approximately 3 million deaths worldwide in 2012 [3]. Fossil fuel combustion is a major source of ambient air pollution: power generation and land traffic alone are estimated to have accounted, for example, for more than half of the approximately 55,000 premature deaths in the United States attributed to ambient air pollution in 2010 [4].

Health risks from air pollution vary greatly across countries, with the highest premature death rates generally occurring in Eastern European countries [3]. The distribution of air pollution health risk is quite uneven within countries as well, and exposure is often inversely related to socioeconomic status. In the US, including California, studies have documented that ethnic minority and lower-income communities bear disproportionate air pollution burdens [5–8].

These patterns suggest that carbon pricing and other climate policies that reduce fossil fuel combustion could generate improvements in air quality that will benefit disadvantaged communities. Nevertheless, environmental justice groups in California have opposed the state’s cap-and-trade program, expressing concern that it could lead to increased co-pollutant emissions in minority and low-income communities [9].

## California’s experience

California’s Global Warming Solutions Act of 2006 required the California Air Resources Board to develop a set of policies to reduce the state’s GHG emissions to the 1990 level by 2020. Compliance obligations for industrial facilities under the cap-and-trade program went into effect in January 2013. Covered sectors include electrical generation, manufacturing, cement production, and oil and gas production and supply. The majority of the state’s carbon mitigation has been attributable to other “complementary measures,” such as renewable portfolio standards for electric utilities and low carbon fuel standards for automobiles; the cap-and-trade program accounts for less than one-third of total mitigation [10,11]. The study by Morello-Frosch and colleagues focuses on the large point-source emitters targeted by the cap-and-trade program, but the emission trends for these facilities are the joint outcome of both types of policies.

Two features of California’s cap-and-trade program have substantially reduced its impact on co-pollutant emissions. The first is the practice known as “regulatory shuffling,” whereby contracts for electricity delivered from other states are modified to replace coal-fired plants with lower carbon-emitting sources. This does not reduce in-state co-pollutant emissions (nor out-of-state emissions if mirrored by a commensurate increase in the coal share of electricity consumed out of state). The second is the provision for “offsets,” whereby part of the mitigation requirement can be met by forestry or other projects intended to reduce atmospheric GHG levels. It is not surprising, therefore, that emissions of both GHGs and co-pollutants increased at many facilities.

Morello-Frosch and colleagues examined the Census block groups that lie within 2.5 miles of at least one industrial facility and, by this proximity indicator, were at relatively high risk of exposure to co-pollutants. Residents of these neighborhoods were more likely to be ethnic minority, poor, and less educated than Californians who live farther than 2.5 miles from any facility (see Table 1 of [2]).

The most noteworthy contribution of the study lies in its analysis of the correlation between socioeconomic status and changes in emissions after implementation of the cap-and-trade program. Of the population living within 2.5 miles of emitters, roughly 60% experienced declines in net GHG emissions from those facilities, but 40% experienced increases. In the latter group, many also saw increased co-pollutant emissions (Table 3 of [2]). Compared with neighborhoods in which GHG emissions decreased, communities in which they increased had 11 percentage points more ethnic minority people, 7 percentage points more with low education, and were 16 percentage points more likely to be living in a disadvantaged community as defined by CalEnviroScreen (all disparities statistically significant, with *p* < 0.001) (Table 3 of [2]). The disparities were even larger in neighborhoods that experienced increases in PM_2.5_, VOCs, or air toxics as well as GHGs. In multivariate analysis, the percentage of ethnic minority and less educated people were significant and substantive predictors of the likelihood of increases in emissions (Table 4 of [2]).

## Implications

The study by Morello-Frosch and colleagues underscores the importance of monitoring co-pollutant emissions during the implementation of climate policies. An important step to facilitate this would be improved integration of GHG data registries with other pollution databases such as the Toxics Release Inventory and the National Emissions Inventory. At present, the absence of consistent facility identifiers poses a substantial hurdle to analysis of the association between GHGs and co-pollutants, which may be one reason why such studies have been few in number [2,12].

Further research is needed to assess how climate policy design influences the magnitude and distribution of public health co-benefits. California’s cap-and-trade program was extended to transportation fuels in 2015, and co-pollutant impacts in this sector are yet to be analyzed. Similar studies on the impact of the European Union’s Emissions Trading System as well as the world’s largest cap-and-trade program for carbon emissions that is now being introduced in China—where concerns over integrating air quality co-benefits into the policy and about air pollution being adversely affected in specific localities have also been raised—are needed [13].

New legislation signed into law last year will require a further 40% reduction in California’s GHG emissions in the 2020–2030 period. The cap-and-trade program may play a more prominent role in achieving these deeper cuts. In light of Morello-Frosch and colleagues’ findings, it would be prudent to consider policy design features—such as zonal or sectoral systems with “sub-caps” on polluters in priority locations or sectors—designed minimally to avoid increases in co-pollutant emissions in disadvantaged communities or, more ambitiously, to mitigate disparities by obtaining greater co-pollutant emission reductions in overburdened communities [12].

## Abbreviations

GHG: greenhouse gas
PM_2.5_: particulate matter of diameter less than 2.5 micrometers
VOC: volatile organic compound
